# Vitamin D Concentration Among Women with Gynecological Cancers

**DOI:** 10.3390/cancers17121987

**Published:** 2025-06-14

**Authors:** Marcin Adam Zębalski, Patrycja Zębalska, Aleksandra Krzywon, Krzysztof Nowosielski

**Affiliations:** 1 Department of Gynecology, Obstetrics and Gynecological Oncology, University Clinical Center of the Medical University of Silesia, 40-752 Katowice, Poland; marcin.zebalski@sum.edu.pl (M.A.Z.); ginpol@uck.katowice.pl (P.Z.); 2 Department of Biostatistics and Bioinformatics, Maria Sklodowska-Curie National Research Institute of Oncology, Gliwice Branch, 44-102 Gliwice, Poland

**Keywords:** vitamin D, oncology, ovarian cancer, cervical cancer, endometrial cancer

## Abstract

**Background:** Although vitamin D supplementation is simple, inexpensive, and safe, vitamin D deficiency remains widespread, especially in developing communities. The aim of our study was to assess vitamin D levels among patients with gynecological cancers and compare them with those in patients with benign tumors living in rural and urban areas. **Methods**: This is a clinical retrospective study covering data analysis from March 2021 to July 2023. A total of 686 patients with uterine or ovarian tumors were analyzed. An electrochemiluminescence immunoassay method was used to assess vitamin D concentrations. Other laboratory blood tests were also performed on the admission day. **Results**: A significant reduction in vitamin D levels in oncological vs. non-oncological patients (median 23 (17, 33) ng/mL vs. 28 [21, 36] ng/mL, *p* < 0.001) was observed. The lowest vitamin D concentration was found in patients with ovarian cancer (median 22 (16, 32) ng/mL), followed by those with endometrial cancer and cervical cancer—median 24 (18, 35) ng/mL and 26 (20, 31) ng/mL, respectively). We found no differences in the vitamin D concentration between various histopathological types of ovarian cancers (*p* = 0.07). No correlation between the vitamin D concentration and age (r = 0.03, *p* > 0.05) was noted. A negligible negative correlation between vitamin D levels and BMI was observed (r = −0.095, *p* = 0.03). Additionally, those living in cities had a significantly reduced vitamin D concentration compared to those living in rural areas. No significant differences were demonstrated in vitamin D concentrations between malignant and benign tumors among patients living in rural areas (*p* = 0.17). **Conclusions**: Gynecological oncology patients have significantly lower vitamin D levels compared to non-oncological patients. In our patient population, ovarian and endometrial cancers were frequently associated with vitamin D deficiency. While this observation does not establish causation, it highlights the potential value of monitoring vitamin D levels and addressing deficiencies as part of broader cancer prevention and management strategies.

## Simple Summary

Vitamin D deficiency is widespread, and the incidence of cancer continues to rise. Numerous studies have shown a link between vitamin D deficiency and cancer incidence. However, no studies to date have examined the association between vitamin D status and distinct histopathological types of gynecological cancers. Ensuring proper vitamin D levels may serve as a protective factor against gynecological malignancies.

## 1. Introduction

Calcitriol, the active form of vitamin D3, is a fat-soluble vitamin mostly produced in the human body through exposure to sunlight. Under ultraviolet B (UVB) radiation, 7-dehydrocholesterol in the skin is converted to cholecalciferol, which is then hydroxylated in the liver to form 25-hydroxyvitamin D3, and subsequently in the kidneys to 1,25-dihydroxyvitamin D3, the main active hormone. The secondary source of vitamin D3 is dietary intake, mainly from fish, butter, eggs and vegetable oils [1].

Vitamin D is mainly responsible for calcium and phosphate metabolism. Nevertheless, some authors claim it should be called a hormone because it is responsible for many regulatory processes in the body, including the expression of many genes that may play a role in the pathogenesis of cancer and the modulation of the immune system [2]. In addition to its anti-cancer effect, vitamin D3 is believed to have a positive effect on the cardiovascular system, reducing the risk of cardiovascular events, and positively influencing the immune system and carbohydrate metabolism [3,4].

In recent years, more attention has been paid to the anti-cancer effect of vitamin D. Many studies have noted a relationship between low vitamin D3 concentrations and a higher incidence of cancer [2,5]. It has been observed that vitamin D may have a protective effect on gynecological cancers including ovarian, endometrial and cervical cancer, as well as many other types of cancer, such as colon, breast, prostate, melanoma, and some leukemias and lymphomas [1,3,6].

The effect of vitamin D3 on the etiopathogenesis of gynecological cancers is not fully elucidated. However, many studies postulate that adequate vitamin D3 concentrations may have a protective effect against ovarian cancer [1,6,7,8,9,10,11,12,13,14] and cervical cancer [15,16,17,18,19]. The evidence for an association between vitamin D and endometrial cancer is not fully consistent [8].

Vitamin D3 has been shown to inhibit the proliferation of cancer cells in vitro [8]. The inhibition of cancer cell division is achieved by blocking cell cycle checkpoints, reducing transcription factors, and influencing the epithelial–mesenchymal transition, which results in the inhibition of cancer cell migration and invasion. Moreover, vitamin D reduces the level of telomerase in ovarian cancer cells, which translates into the induction of apoptosis [12,20,21,22,23,24,25]. The result is the inhibition of the growth of many ovarian cancer cell lines [25]. In vivo and in vitro studies have also demonstrated the effect of vitamin D on inhibiting the metastasis of ovarian cancer to the omentum [26].

The aim of this study was to evaluate the vitamin D concentration in patients with gynecological cancers compared to non-oncological patients and to assess the vitamin D concentration depending on the histopathological type of the tumor.

## 2. Materials and Methods

### 2.1. Patients

This single-center, retrospective analytical study assesses vitamin D levels in patients who underwent surgery at the Department of Obstetrics, Gynecology, and Oncologic Gynecology in Katowice, Poland, due to gynecological malignant tumors and oncologically suspicious uterine and adnexal tumors.

Vitamin D3 levels were measured in all patients with gynecological cancers and those with suspected ovarian or uterine tumors. Blood samples for vitamin D assessment were collected in the morning as part of routine preoperative laboratory testing, on the day of hospital admission (i.e., the day before the scheduled surgery). The total serum vitamin D levels were measured using an electrochemiluminescence immunoassay on the Cobas e402 (Roche, Basel, Switzerland).

The exclusion criteria included patients admitted to the hospital for purposes other than surgery due to gynecological cancer or its suspicion.

For the correct serum levels of vitamin D, we used a range between 30 and 100 ng/mL in accordance with applicable standards [27,28,29].

### 2.2. Statistical Analysis

Medians with interquartile ranges (25% to 75%, IQR) and means with standard deviations were provided for continuous variables. The Wilcoxon rank sum test (for two groups), the Kruskal–Wallis H test (for more than two groups) and the post hoc Dunn test were used for continuous variables. A one-sample Wilcoxon signed rank test was performed for the changes measured between two time points. The Spearman correlation coefficient was used to calculate a measure of correlation according to the following classification: 0.0 ≤ |r| < 0.1 negligible correlation, 0.1 ≤ |r| ≤ 0.39 weak correlation, 0.4 ≤ |r| ≤ 0.69 moderate correlation, 0.7 ≤ |r| ≤ 0.89 strong correlation, 0.9 ≤ r ≤ 1 very strong correlation. Statistical significance was defined as a two-sided *p*-value < 0.05. All analyses were performed using the R environment for statistical computing (version 4.0.1, R Foundation for Statistical Computing, Vienna, Austria, http://www.r-project.org, accessed on 6 June 2020).

## 3. Results

### 3.1. Patients

In this study, we analyzed the medical records of 686 patients hospitalized between March 2021 and July 2023 at the Department of Obstetrics, Gynecology, and Oncologic Gynecology in Katowice, Poland. All patients were white, Polish-born women.

We divided our cohort (*n* = 686 patients) into oncological patients with a histopathologically confirmed malignancy postoperatively (Group A—oncological patients; *n* = 472, 69%) and non-oncological patients (Group B—non-oncological patients, *n* = 214; 31%). The basic characteristics of the study participants are summarized in Table 1. Both groups were comparable in terms of age (*p* > 0.05), BMI (*p* > 0.05), comorbidities like diabetes, hypertension and ischemic heart disease (*p* > 0.05), smoking status (*p* > 0.05), the frequency of oral contraceptive use and urban residency (*p* > 0.05).

Among oncological patients, 283 (60%) were patients with ovarian cancer, 135 (29%) had endometrial cancer and 54 (11%) had cervical cancer. Among non-oncological patients, 52 (24.3%) had uterine fibroid and 162 (75.7%) had benign ovarian lesions. Detailed histopathological results are presented in Table 2.

### 3.2. Surgery

Of the total patients, 266 patients (39%) underwent laparotomy, 404 (59%) underwent laparoscopy, and 16 patients (2%) were disqualified from surgery due to their poor general condition and failure to meet anesthesia qualifications. In patients who were not eligible for surgery, the diagnosis of cancer was either confirmed or excluded through dilation and curettage (D&C), cervical biopsies, or CT-guided biopsy for histopathological examination.

### 3.3. Vitamin D Concentration

**Figure 1:**
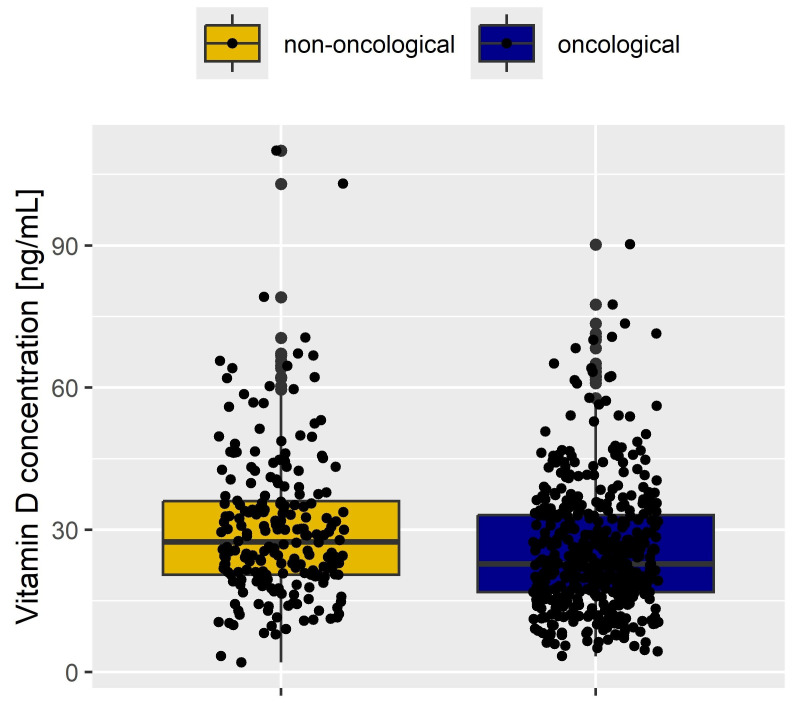
Comparison of vitamin D levels in oncological and non-oncological patients.

We have shown that the median concentration of vitamin D is significantly reduced in oncological patients compared to non-oncological patients (23 (17, 33) ng/mL vs. 28 (21, 36) ng/mL, *p* < 0.001)—Figure 1.

Vitamin D deficiency was observed in 69% of oncological patients compared to 55% of non-oncological patients (*p* < 0.001).

Among oncological patients, those with ovarian cancer had the lowest median vitamin D concentration (22 (16, 32) ng/mL), followed by patients with endometrial cancer (24 (18, 35) ng/mL) and cervical cancer (26 (20, 31) ng/mL). Compared to non-oncological patients, significantly lower levels were found in ovarian and endometrial cancer patients (*p* < 0.001), whereas the difference for cervical cancer was not statistically significant (*p* = 0.1) (Table 3).

**Figure 2:**
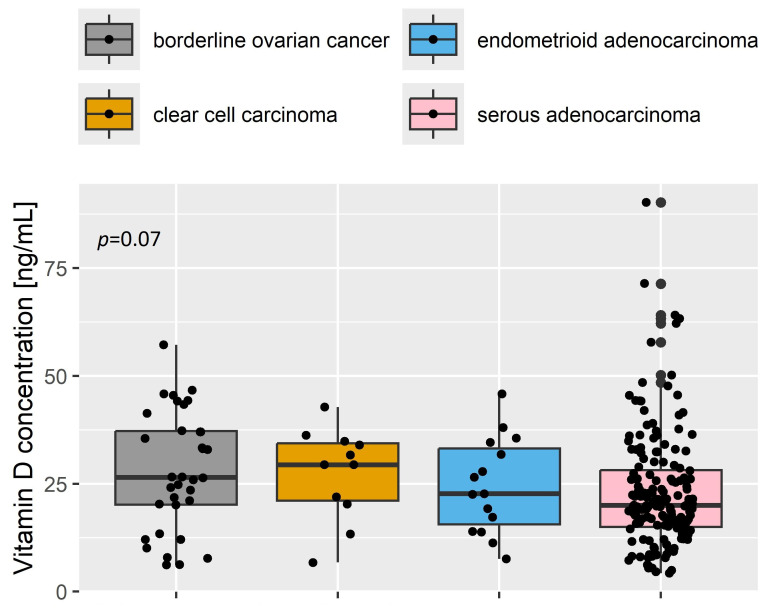
Vitamin D concentration in individual histopathological types of ovarian cancer.

Among ovarian cancer patients, the most common ovarian cancers were serous adenocarcinoma (169 patients, 60%), endometroid adenocarcinoma (15 patients, 5%), clear cell carcinoma (11 patients, 4%), and borderline tumors (34 patients, 12%). We assessed the vitamin D levels in patients with various histopathological types of ovarian cancer. Due to the small sample size, patients with mucinous adenocarcinoma (5 patients, 2%), undifferentiated carcinoma (3 patients, 1%) and other rare subtypes were excluded from further analysis. No significant differences (*p* = 0.07) in vitamin D concentrations were observed between various histopathological types of ovarian malignant tumors, as shown in Figure 2.

No correlation was found between vitamin D concentrations and age (r = 0.03, *p* > 0.05). A negligible negative correlation was observed with BMI (r = −0.095, *p* = 0.03).

No significant difference in vitamin D levels was found between rural and urban residents overall (median 26 (20, 33) ng/mL vs. 24 (18, 34) ng/mL, *p* > 0.05). However, when stratified by disease status, urban oncological patients had significantly lower vitamin D levels than their non-oncological counterparts (*p* < 0.001), a difference not observed in rural patients (*p* = 0.11) (Table 4).

Since 2021, the Department of Obstetrics, Gynecology, and Oncologic Gynecology in Katowice has implemented a prehabilitation program, which includes correcting vitamin D deficiency preoperatively. Among the 472 oncological patients, 110 participated in this program. A significant increase in vitamin D concentrations was observed during the prehabilitation period (median increase: 8 (1, 18) ng/mL, *p* < 0.001 Table 5).

## 4. Discussion

This retrospective analysis revealed significantly lower vitamin D levels in oncological compared to non-oncological patients, especially among those with ovarian and endometrial cancer. No significant differences were found among various histological types of gynecological cancers. These findings align with the existing literature, which frequently reports vitamin D deficiency in cancer patients.

Vitamin D deficiency is widespread, especially in developed countries, and is strongly influenced by geographic latitude. Research shows that deficiency (25-OH-D < 20 ng/mL) affects around 40% of Europeans and 22% of Americans and Canadians [30,31].

The primary source of vitamin D is cutaneous synthesis via UVB radiation. Dietary intake and supplementation are secondary [21,32,33,34]. Skin synthesis is significantly more effective, as shown by Punnonen et al., who found comparable levels of fat-soluble vitamins A and E between Finnish and Floridian women but double the vitamin D levels in the latter [34].

There are many factors that limit the maintenance of appropriate vitamin D concentrations. One of the main factors is the geographical latitude inhabited by a given population. In addition, a low number of sunny days per year and a lack of skin exposure to sunlight further contribute to Vitamin D deficiency. The use of sunscreen, which limits vitamin D synthesis despite sun exposure, is another factor. Moreover, a patient’s BMI, skin melanin concentration, and skin pigmentation also influence vitamin D synthesis [3,5,21,31,35,36,37].

In 1980, the Garland brothers noticed a link between living at higher latitudes, vitamin D deficiency, and an increased risk of malignant tumors [38]. Since then, numerous studies have supported a link between sunlight exposure, vitamin D levels, and cancer occurrence [1,3,5,6,7,8,9,10,11,12,13,14,15,38,39,40,41,42,43,44]. In our study, we found significantly lower vitamin D levels in patients with ovarian cancer compared to those with benign lesions. Similar findings were reported by Bakhru et al. and Web et al., who found significantly lower vitamin D concentrations in ovarian cancer patients compared to healthy individuals [44,45]. Additionally, higher vitamin D levels in ovarian cancer patients were associated with longer survival [7,45,46]. A meta-analysis by Keum et al. showed that vitamin D3 supplementation was associated with a 13% reduction in cancer mortality [47].

However, not all studies confirm a link between vitamin D and the incidence of ovarian cancer [42,48,49,50,51,52]. One Mendelian randomization study found little evidence of a linear association between the genetic determinants of circulating vitamin D and ovarian cancer risk, although the possibility of a low magnitude having clinically relevant effects could not be ruled out [48]. In a study by Sajo et al., no clear association was found between serum vitamin D deficiency and the risk of ovarian cancer, although the median vitamin D levels were significantly lower in ovarian cancer patients than in cancer-free individuals (33.8 (23.0, 53.0) nmol/L vs. 50.0 (32.5, 93.0) nmol/L). This study was conducted in Africa, where sun exposure is significantly higher than in Europe or the U.S. [49]. A meta-analysis by Xu et al. did not find that vitamin D reduced the risk of ovarian cancer. While important, this study focused primarily on vitamin D and calcium intake, rather than serum vitamin D levels [50]. Similarly, a meta-analysis by Yin et al. found a tentative inverse association between circulating vitamin D and ovarian cancer, but this did not reach statistical significance, and the authors emphasized the need for further research [41].

We also found significantly lower vitamin D concentrations in patients with endometrial cancer compared to non-oncological patients. A relationship was also noted between endometrial cancer risk and geographical latitude, solar radiation exposure, and vitamin D levels [53,54,55]. A meta-analysis by McCullough et al. found no significant association between vitamin D intake and endometrial cancer risk; however, it should be noted that this study evaluated intake rather than circulating levels. As noted by Punnonen et al., diet alone may be an insufficient source of vitamin D—a point also acknowledged in this meta-analysis, which emphasized the need for studies including serum vitamin D levels and sun exposure assessments [34,55]. A nested case–control study by Zeleniuch-Jacquotte et al. also showed no link between circulating vitamin D and endometrial cancer risk [56]. Conversely, Yu et al. showed that dietary vitamin D may mitigate the carcinogenic impact of obesity on the endometrium [57]. Given the limited number of studies, more research is needed to draw firm conclusions.

Vitamin D is also believed to have an inhibitory effect on cervical cancer [58,59,60]. A higher incidence of vitamin D deficiency was observed in patients with cervical intraepithelial neoplasia (CIN) and invasive cervical cancer [16,18,61]. It has been shown that vitamin D may have a protective effect on cervical cancer by reducing the expression of oncogenic proteins and inhibiting the proliferation of cell lines [19,62]. While HPV infection is the primary cause of cervical cancer, vitamin D may influence the HPV life cycle through antiviral effects [18]. A randomized trial by Vahedpoor et al. showed the significant regression of CIN1 lesions over a 6-month period in women treated with vitamin D compared to those without supplementation [16].

To our knowledge, no studies compared vitamin D concentrations across different histopathological types of gynecological cancers. In our study, we did not find that any particular histopathological type, including ovarian cancer, was associated with significantly lower vitamin D levels. However, due to the small number of patients with rare histopathological types, further studies on larger cohorts seem justified.

Most studies to date confirm that populations living in urban areas have lower vitamin D concentrations compared to rural populations [63,64,65,66,67,68,69,70]. However, some studies have reported the opposite, with higher levels in city residents [71]. In our study, we did not find lower vitamin D levels among rural patients compared to urban ones. Notably, we observed significantly lower vitamin D concentrations among urban oncology patients, a trend not seen in rural patients. It should be emphasized that the rural patient group in our study was much smaller, and place of residence was not the primary focus of our investigation.

In clinical practice, attention is paid to the treatment of vitamin D deficiency. In our department, as part of the prehabilitation program for patients scheduled for ovarian cancer surgery, one of the recommended interventions is preoperative vitamin D supplementation, especially in deficient patients [72]. Among those in the program, a significant increase in vitamin D levels was observed during the prehabilitation period. While there is no strong evidence that higher vitamin D levels reduce postoperative complications, supplementation is recommended due to its general health benefits, improved prognosis, and reduced chemotherapy-related side effects [73].

A key strength of our study is the large number of patients included, particularly those with ovarian, endometrial, and cervical cancers. To our knowledge, we are the first to analyze vitamin D levels across various histopathological types of ovarian cancer. Additionally, the patient group was homogeneous in terms of ethnicity and skin color, which is important when studying vitamin D concentrations.

Nonetheless, our study has several important limitations. First, it is retrospective in design, which carries inherent limitations. We lacked data on prior vitamin D supplementation in both groups; however, given the large sample size, we believe any impact on the results is minimal. Additionally, we could not assess lifestyle, physical activity, occupational exposure, or socioeconomic status—all factors that may influence vitamin D levels. Another limitation is that vitamin D was assessed only once, on the day of surgical admission, with no prior measurements available. Finally, although the overall patient number was substantial, the sample size for rare histopathological types of ovarian cancer was too small for statistically significant conclusions.

## 5. Conclusions

In conclusion, our study demonstrated significant vitamin D deficiency among oncology patients compared to non-oncological controls. Specifically, patients with ovarian and endometrial cancer exhibited markedly lower vitamin D levels. While we did not find a relationship between the vitamin D concentration and specific histopathological types of cancer, this is the first analysis of its kind, and further research is needed. Additionally, cancer patients living in urban areas had significantly lower vitamin D levels compared to non-oncological individuals, a pattern not observed among rural residents. In our patient population, ovarian and endometrial cancers were frequently associated with vitamin D deficiency. While this observation does not establish causation, it highlights the potential value of monitoring vitamin D levels and addressing deficiencies as part of broader cancer prevention and management strategies.

## Figures and Tables

**Table 1:** Baseline characteristics of oncological and non-oncological patients.

Characteristic	Oncological Patients (*n* Total = 472)	Non-Oncological Patients (*n* Total = 214)	*p*-Value
Age [years], (SD)	56 (14)	56 (14)	*p* > 0.05
BMI [kg/m^2^], (SD)	27 (6)	27 (6)	*p* > 0.05
City inhabitant, *n*/*n* total (%)	407/472 (86)	189/214 (88)	*p* > 0.05
Diabetes, *n*/*n* total (%)	86/472 (18)	26/214 (12)	*p* > 0.05
Hypertension, *n*/*n* total (%)	197/472 (42)	81/214 (38)	*p* > 0.05
Ischemic heart disease, *n*/*n* total (%)	33/472 (7)	13/214 (6)	*p* > 0.05
Smoking, *n*/*n* total (%)	103/472 (22)	47/214 (22)	*p* > 0.05
Use of oral contraceptives, *n*/*n* total (%)	23/472 (4.8)	13/214 (6.1)	*p* > 0.05

Abbreviations: SD—standard deviation, BMI—body mass index.

**Table 2:** Detailed characteristics and histopathological results of patients in both groups.

Characteristic	Number of Patients
**Oncological patients,** ***n*****/*****n*** **total (%)**	**472/686 (68.8)**
Ovarian cancer, *n*/*n* total of oncological patients (%)	283/472 (60)
Endometrial cancer, *n*/*n* total of oncological patients (%)	135/472 (29)
Cervical cancer, *n*/*n* total of oncological patients (%)	54/472 (11)
**Non-oncological patients,** ***n*****/*****n*** **total (%)**	**214/686 (31.2)**
Uterine fibroid, *n*/*n* total of non-oncologic patients (%)	52/214 (24.3)
Benign ovarian lesions, *n*/*n* total of non-oncologic patients (%)	162/214 (75.7)
Serous adenoma, *n*/*n* total of benign ovarian lesions (%)	22/162 (13.6)
Mucous adenoma, *n*/*n* total of benign ovarian lesions (%)	12/162 (7.4)
Teratoma, *n*/*n* total of benign ovarian lesions (%)	11/162 (6.8)
Fibroma, *n*/*n* total of benign ovarian lesions (%)	3/162 (1.9)
Thecoma, *n*/*n* total of benign ovarian lesions (%)	2/162 (1.2)
Benign Brenner tumor, *n*/*n* total of benign ovarian lesions (%)	3/162 (1.9)
Sertoli-Leydig cell tumor, *n*/*n* total of benign ovarian lesions (%)	2/162 (1.2)
Endometrial cyst, *n*/*n* total of benign ovarian lesions (%)	16/162 (9.9)
Hemorrhagic cyst, *n*/*n* total of benign ovarian lesions (%)	14/162 (8.6)
Serous cyst, *n*/*n* total of benign ovarian lesions (%)	77/162 (47.5)

**Table 3:** Comparison of vitamin D concentrations in ovarian, endometrial and cervical cancers compared to non-oncological patients.

Type of Cancer vs. Non-Oncological Patients	Number of Patients	Vitamin D Concentration (Median [IQR], ng/mL	*p*-Value
Ovarian cancer vs. non-oncological patients	283	22 (16, 32)	
	vs.	vs.	<0.01
	214	28 (21, 36)	
Endometrial cancer vs. non-oncological patients	135	24 (18, 35)	
	vs. 214	vs. 28 (21, 36)	<0.01
Cervical cancer vs. non-oncological patients	54	26 (20, 31)	
	vs. 214	vs. 28 (21, 36)	0.1

Abbreviations: IQR—interquartile range.

**Table 4:** Median vitamin D concentrations by residence and disease status.

Characteristic	Number of Patients	Non-Oncological Patients from Countryside, *n* = 25	Oncological Patients from Countryside, *n* = 65	Non-Oncological Patients from City, *n* = 189	Oncological Patients from City, *n* = 408	*p*-Value
Vitamin D concentration, median [IQR] (ng/mL)	686	29 (22, 36)	24 (19, 32)	27 (21, 36)	23 (17, 33)	<0.001

Abbreviations: IQR—interquartile range.

**Table 5:** Change in vitamin D concentration during prehabilitation.

Characteristic	Beginning of Prehabilitation Program	End of Prehabilitation Program	Change *	*p*-Value
Vitamin D concentration, median [IQR] (ng/mL)	23 (17, 33)	35 (28, 46)	8 (1, 18)	<0.001

Abbreviations: IQR—interquartile range. * The difference in vitamin D concentration during prehabilitation program.

## Data Availability

Data are contained within the article.
